# Ultrasound measurements for evaluation of changes in upper airway during anaesthesia induction and prediction difficult laryngoscopy: a prospective observational study

**DOI:** 10.1038/s41598-022-21695-2

**Published:** 2022-11-03

**Authors:** Xia Wang, Yong Wang, Zhen-Wei Zheng, Yu-Rui Liu, Wu-Hua Ma

**Affiliations:** 1 First Clinical Medical College, Guangzhou University of Chinese Medicine, Guangzhou, China; 2 Department of Anaesthesia, The First Affiliated Hospital of Guangzhou University of Chinese Medicine, Guangzhou, China; 3 Department of Anaesthesiology, Second Affiliated Hospital of Shantou University Medical College, Shantou, China

**Keywords:** Oral anatomy, Clinical trial design, Ultrasonography

## Abstract

Upper airway collapsibility after anaesthesia induction may be associated with unpredictable difficult airway. However, most works on airway anatomy are tended to morphological description before anaesthesia. This study aimed to evaluate the changes of upper airway after anaesthesia induction and using pre-anesthetic ultrasound measurements to predict Difficult Laryngoscopy (DL). We included 104 eligible subjects with complete data, who were performed tracheal intubations under general anaesthesia in the study. The upper airway changes before and after anaesthesia induction were determined by seven neck ultrasound measurements, included as follow: (1) Distance from skin to under surface of Tongue (DT), (2) Thickness of the thickest part of Tongue body (TT), (3) Hyoid Mental Distance (HMD), (4) Depth of Hyoid (DH), (5) Width of Hyoid (WH), (6) Distance from Skin to Epiglottis (DSE), (7) Depth of the anterior combination of the Vocal Cords (DVC). DL was evaluated with Cormack–Lehane (CL). Data regarding HMD [from 45.3 (42.4–48.5) to 41.1 (38.5–44.9) mm], DH [from 8.7 (6.6–10.9) to 7.0 (5.3–9.1) mm], DSE [from 20.1 (16.6–22.5) to 19.5 (16.5–21.6) mm] and the DVC [from 7.1 (5.7–8.3) to 6.8 (5.7–7.9) mm] were decreased (*P* < 0.05), while the DT [from 15.9 (13.1–18.4) to 17.4 (14.5–19.8) mm] was increased (*P* > 0.05) after anaesthesia induction. Additionally, when cut-off value of DSE was 21.25 mm before anaesthesia, it may be better predicted to DL [sensitivity 80.0% (95% CI: 60.7–91.6%) and specificity 83.8% (95% CI: 73.0–91.0%)]. The upper airway after induction showed the propensity of collapsibility by ultrasound measurements. Compared with other indicators, the DSE assessed by ultrasound might be considered to a valuable predictor of DL.

Trial registration: The study was registered in ClinicalTrials.gov on 23th Jan 2019, ChiCTR1900021123.

## Introduction

Difficult airway management is one of the most relevant issues for practicing emergency physicians, intensivists, and anaesthesiologists, since airway obstruction in an unexpected patient can lead to brain damage even death^1–3^. It is well known that increasing mortality of anaesthesia was connected with collapsibility and obstruction of upper airway^4,5^. Deficiency of pharyngeal patency may be associated with changes in neck anatomy after anaesthesia induction, because local electromyographic activity abruptly decreases to a nadir^6,7^. Correct management of airway collapsibility during anaesthesia induction largely depends on an in-depth knowledge of the anatomic changes of upper airway^8^, because the changes of upper airway after anaesthesia are complicated. However, an accurate evaluation of the airway collapsibility may not be obtained with neck parameters before anaesthesia, and there are less sufficient scientific evidences for detection of upper airway changes after induction of anaesthesia. Robust, prospective evidences were needed to identify the changes of upper airway and predict difficult airway during anaesthesia induction.

Accordingly, point-of-care ultrasound is an effective, non-invasive and widely available tool, which can detect morphologic changes in upper airway with no radiation and little patient preparations^9–11^. It was hypothesized that the airway collapsibility could be described by ultrasonic anatomical parameters in patients under anaesthesia induction(primary outcome) and pre-anesthetic ultrasound measurements in upper airway could predict difficult airway(secondary outcome).

## Methods

The study was designed as a prospective observational study. Ethical approval for this study was provided by the ethics committee of the first affiliated hospital of Guangzhou University of Chinese Medicine (NO.ZYYECK (2018) 041), and this trial was registered in the clinical trial registry of China (ChiCTR1900021123). Informed written consent was obtained. The study was conducted in accordance with the guidelines provided by the World Medical Association Declaration of Helsinki on Ethical Principles for Medical Research Involving Humans, and we confirmed that all experiments were performed in accordance with relevant guidelines and regulations. We recruited patients who had undergone elective surgery with general anaesthesia in the First Affiliated Hospital of Guangzhou University of Chinese Medicine between February 2019 and January 2020. The patients presented with American Society of Anaesthesiologists (ASA) physical status I or II and were aged between 18 and 70 years. Inclusion and exclusion criteria were shown in Table 1.

**Table 1:** Trial inclusion and exclusion criteria.

Inclusion criteria	Exclusion criteria
Age: 18–70 years	BMI > 35 kg·m^−2^, pregnant women, and parturients
ASA grade I or II	Patients with predicted difficult airway
Undergoing elective surgery with general anaesthesia	Patients with abnormal pharynx or anatomy, severe cardiovascular or cardiopulmonary diseases, hepatic or renal dysfunction, preoperative, coagulation disorders
Consent to participate	Refusal to participate

*BMI* body mass index, *ASA* American Society of Anesthesiologists.

Random number table was used to select two eligible patients every day. To reduce the risk of bias, only one anaesthetist using ultrasound device (Navi series, Shenzhen Wisonic Medical Technologies, Shenzhen, China) proficiently was responsible for measurement and data record. Preoperative evaluation was performed the day before surgery, and informed consent was obtained from each subject. The demographic characteristics and clinical screening tests including gender, BMI, mouth opening, thyromental distance, neck circumference and modified Mallampati grade were collected. After routine fasting (no solids ≥ 8 h; no water ≥ 2 h), patients were sent to operating rooms (OR) and positioned supine on the operating tables with their heads in sniffing position^7,12^. The ECG, SpO_2_, noninvasive blood pressure, capnography and accelerometric neuromuscular monitoring of train-of-four (TOF) were employed.

**Figure 1:**
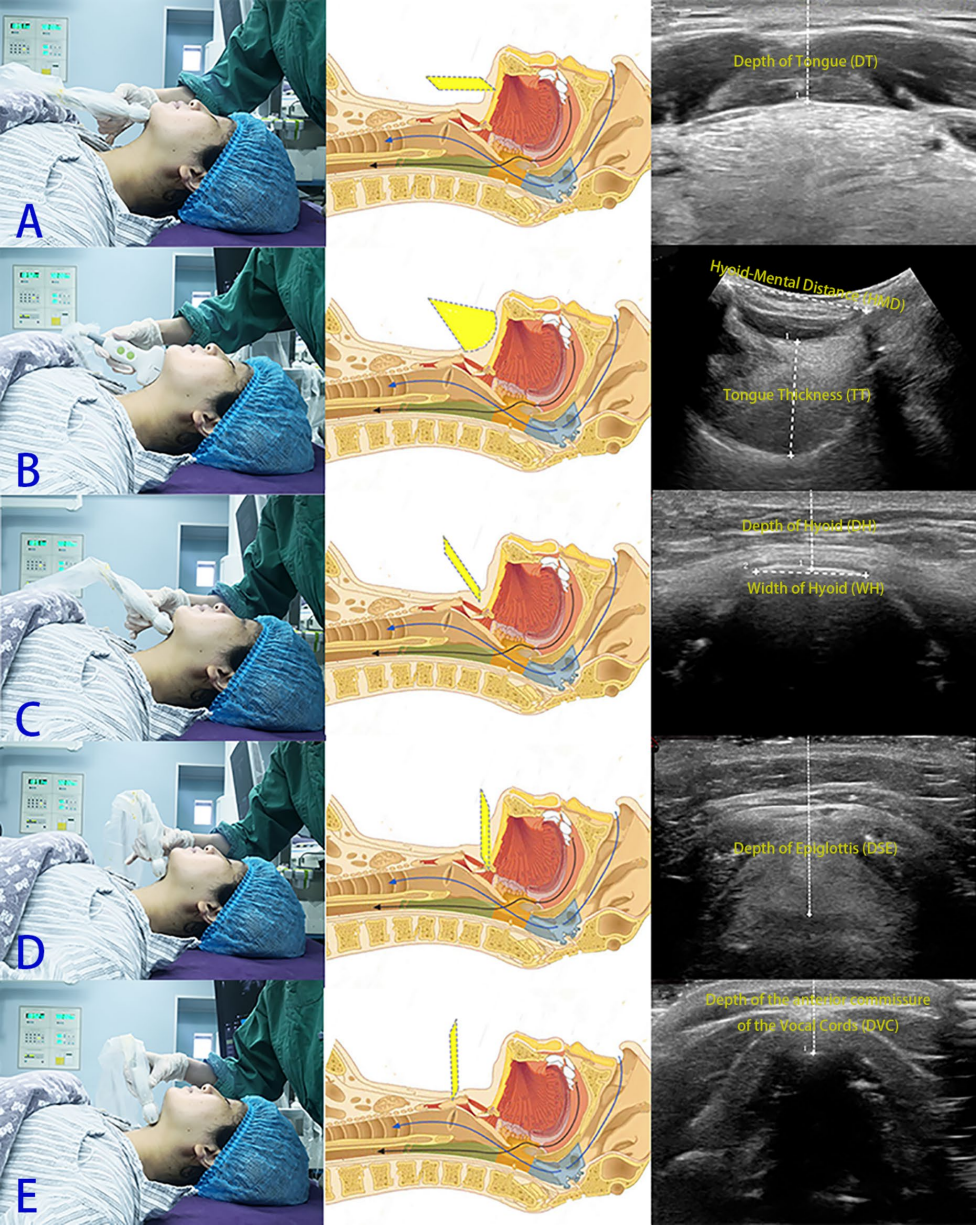
Patient positioning and transducer placement for measuring (left), anatomical (middle) and ultrasonography views (right) at the level of the tongue surface (**A**), tongue body (**B**), hyoid (**C**), epiglottis (**D**) and vocal cords (**E**), before anaesthetic induction. Consent to publish the figure was obtained from the patient.

To compare the changes in upper airway during induction of anaesthesia and assess DL, a cranio-caudal scan of the neck with the probe placed in the transverse and longitudinal axis was performed and 7 kinds of distances from 5 ultrasound images were measured as follows:

1. Distance from skin to under surface of Tongue (Depth of Tongue, DT), Fig. 1A^13^.

(2) Thickness of the thickest part of Tongue body (Tongue Thickness, TT) and Hyoid-Mental Distance (HMD), Fig. 1B^14,15^.

(3) Distance from Hyoid to skin (Depth of Hyoid, DH) and the Width of Hyoid (WH), Fig. 1C^14^.

(4) Distance from Skin to Epiglottis (Depth of Epiglottis, DSE), Fig. 1D^16^.

(5) Depth of the anterior commissure of the Vocal Cords (DVC), Fig. 1E^14^. The images had been stored on the hard drive of the ultrasound device (Fig. 1).

To obtain the upper airway data after induction in safety, the processes of ultrasound measurements and face mask ventilation were carried out alternately every 3 min. The general anaesthesia was performed by two attending anaesthetists in a standard protocol with a combination of 0.003 to 0.004 mg·kg^−1^ fentanyl, 0.1 mg·kg^−1^ vecuronium, and 1 to 2 mg·kg^−1^ propofol. Depth of anaesthesia was measured by a Narcotrend-Compact monitor (MT MonitorTechnik GmbH & Co.KG, Helsinki, Germany). Pre-oxygenation were lasting for 3 min through a facemask with 100% oxygen (5–6 L·min^−1^). Face mask ventilation was continuously administered to the patient for 5 min till the anaesthetic plane was deep enough (Narcotrend D, train-of-four, TOF ¼ 0). Then, face mask was removed and the same ultrasound device was used to quickly measure the seven kinds of distances of upper airway above when patients were anaesthetised. SpO_2_ changes should be paid more attention during measurement. The process would be stopped if SpO_2_ < 93%. Face mask ventilation was performed until SpO_2_ increased. All procedures would be completed in the duration of SpO_2_ > 93%. If an unpredicted emergency airway was encountered, anaesthetist would instantly asked for help from the airway management team. A non-invasive airway tool or method such as a laryngeal mask airway (LMA) should be used to ensure ventilation. If ventilation was failed, a surgical airway must immediately be established to rescue the patient’s life^3^.

Once all measurements came to an end, face mask ventilation was performed for 5 min again. The conventional direct laryngoscopy with a Macintosh blade was performed to evaluate the Cormack–Lehane grades (CL grades) by another attending anaesthetist who was blinded to the results of the previous ultrasound data. According to a modified version of the CL classification system^17,18^, the patients were designated as Non-Difficult Laryngoscopy group (NDL, CL grades < 2b) and Difficult Laryngoscopy group (DL, CL grades ≥ 2b), respectively^19^.

Alveolar recruitment manoeuvres were performed immediately after orotracheal intubation. Mechanical ventilation was carried out with tidal volume of 8–10 ml·kg^−1^, respiration rate of 10–12 times·min^−1^, and PetCO_2_ of 30–35 mmHg. Subsequent anaesthesia maintenance and surgery were performed as usual.

### Outcomes

The primary outcomes were the changes of the seven neck ultrasound measurements in upper airway before and after anaesthetic induction. The ultrasound measurement was used to evaluate the seven tissue structures: DT, TT, HMD, DH, WH, DSE and DVC. The secondary outcomes included laryngoscopy results of CL grades (early prediction for difficult airway) and correlation between CL grades and each indicator (clinical screening tests and ultrasound measurement data).

### Statistical analysis

According to the literature^16^, the DSE measured by ultrasound might be used to predict DL. Therefore, a pilot study of 20 subjects was carried out to estimate sample size. The change in DSE before and after anaesthesia induction was 0.22 cm ± 0.55 (mean ± standard) in pilot study. Furthermore, the ratio between NDL and DL groups was 3:1 (non-difficult: difficult = 3:1). Pass 11.0 software showed that at least 100 patients would be necessary to obtain statistically significant differences between two groups, when accepting an alpha error of 0.05 and a beta error of 0.20.

The IBM Statistical Package for the Social Sciences (SPSS) V.25.0.was used for data analysis. The measurement data were presented as the means ± standard deviation or median (*P*_25_–*P*_75_), and the categorical variable data were presented as numbers. The Kolmogorov–Smirnov test was used to verify the normality of all ultrasound measurement data. Mann–Whitney U test or χ^2^ was used for variables comparison of demographic characteristics and clinical screening tests. According to distribution sets, changes before and after anaesthesia induction were analyzed by the Wilcoxon rank test. The Medcalc software was used for comparison of the area under (AUC) the receiver-operating characteristic curves (ROC curves) determined the correlation between each indicator and the CL grade, included clinical screening tests and perioperative sonographic data. The Youden index (the maximal difference between sensitivity and 1-specificity) was used to determine the optimal cut-off scores for significant indices, then, sensitivity, specificity, positive predictive value (PPV) and negative predictive value (NPV) of the predictive indicators were calculated by QuickCalcs software. For all analyses, *P* value < 0.05 would be considered significantly.

### Ethics approval

This study was performed in line with the principles of the Declaration of Helsinki. Approval was granted by the ethics committee of the first affiliated hospital of Guangzhou University of Chinese Medicine (NO.ZYYECK (2018) 041), and this trial was registered in the clinical trial registry of China (ChiCTR1900021123).

### Consent to participate

Informed consent was obtained from all individual participants included in the study.

## Results

**Figure 2:**
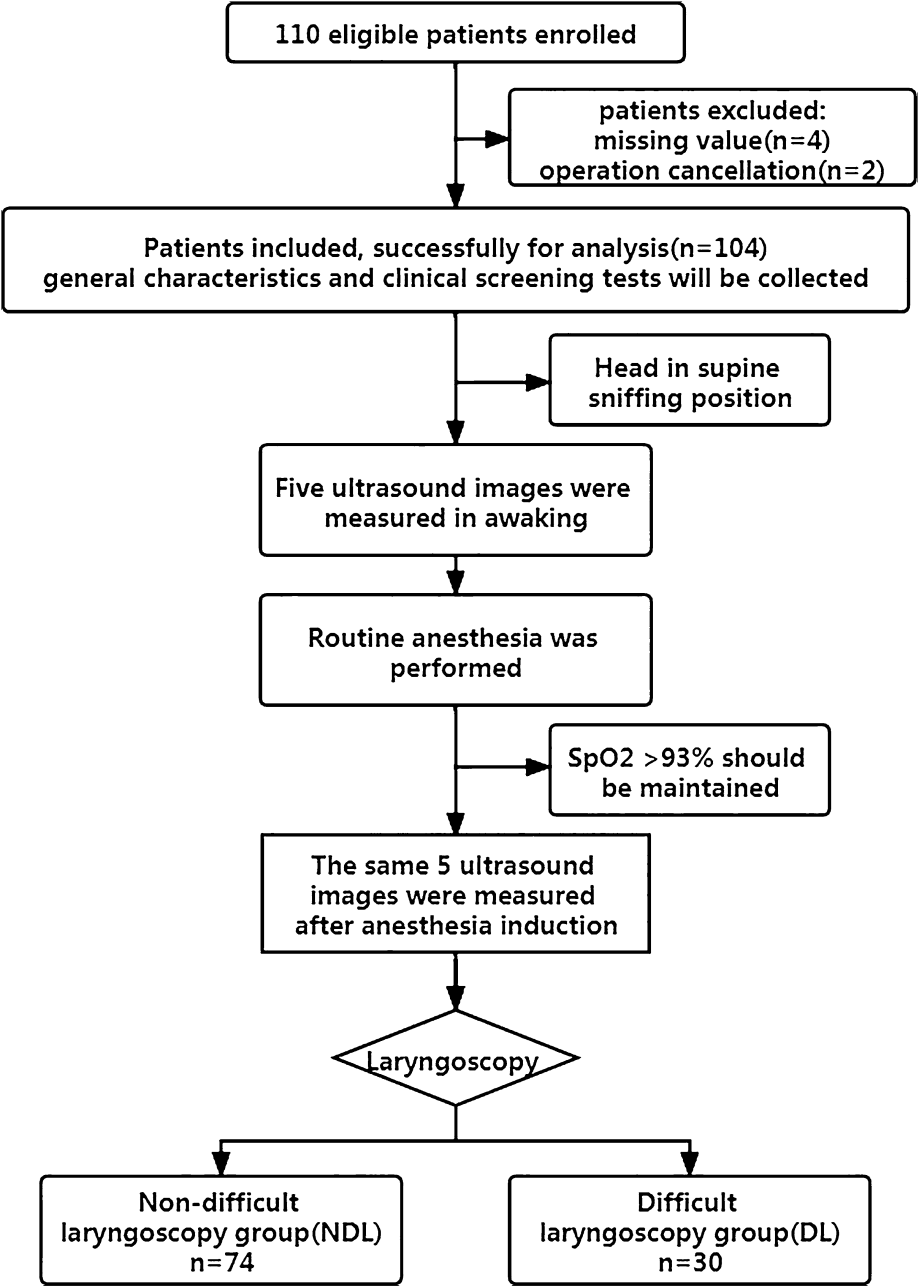
Flow chart of the study.

A total of 110 patients were enrolled. Important missing values occurred in four patients, and two patients were excluded because of cancellation of the operation. Thus, 104 subjects with complete data were successfully included for analysis in this study. The flowchart of this trial was provided in Fig. 2.

There were 48 men (46.15%) and 56 women (53.85%). Participant characteristics are described in Table 2.

**Table 2:** Participant characteristics in two groups.

Variables	DL group (n = 30)	NDL group (n = 74)	*P* value^a^
Sex (male/female, n)	14/16	34/40	0.847
Age (years)	46.7 ± 10.6	45.3 ± 13.0	0.704
ASA grade (I/II)	16/14	34/40	0.495
BMI (kg·m^−2^)	24.2 ± 5.1	21.8 ± 3.0	0.021*
Mouth opening (cm)	4.1 ± 0.8	4.2 ± 0.5	0.309
Thyromental distance (cm)	6.8 ± 0.9	7.4 ± 1.0	0.023*
Neck circumference (cm)	36.2 ± 3.2	33.7 ± 2.6	0.001*
Modified Mallampati grade ≥ 3 (yes/no, n)	15/15	64/10	0.001*
DT (mm)	15.8 (13.6–18.5)	16.1 (13.0–18.4)	0.903
TT (mm)	44.1 (41.3–46.7)	42.0 (39.6–45.5)	0.071
HMD (mm)	44.7 (42.7–47.2)	45.5 (42.3–50.2)	0.275
DH (mm)	8.65 (6.85–10.6)	8.65 (6.60–11.2)	0.929
WH (mm)	11.5 (10.0–13.2)	10.7 (9.75–12.6)	0.251
DSE (mm)	23.4 (21.5–25.7)	18.7 (15.6–20.9)	0.001*
DVC (mm)	7.15 (5.15–8.23)	7.10 (5.80–8.63)	0.696

*NDL* non-difficult laryngoscopy, *DL* difficult laryngoscopy, *ASA* American Society of Anesthesiologists, *BMI* body mass index, *DT* distance from skin to under surface of tongue, *TT* thickness of the thickest part of tongue body, *HMD* hyoid mental distance, *DH* depth of hyoid, *WH* width of hyoid, *DSE* distance from skin to epiglottis, *DVC* depth of the anterior combination of the vocal cords.

Data are shown as the means ± SD deviation and median (*P*_25_–*P*_75_) or numbers.

*P value < 0.05.

^a^Patient characteristics were analyzed by Mann–Whitney U test for continuous variables and χ^2^ test for categorical variables.

We sequentially obtained 5 ultrasound views in the neck. Compared with the data before anaesthesia, the HMD, DH, DSE and the DVC were decreased (*P* < 0.05), while the DT (*P* < 0.05) was increased after anaesthesia induction. All data are shown in Table 3.

**Table 3:** Sonographic variables before and after anaesthesia induction and pair wise comparisons.

Sonographic variables	Before anaesthesia (n = 104)	After anaesthesia (n = 104)	*P* value^b^
DT (mm)	15.9 (13.1–18.4)	17.4 (14.5–19.8)	0.001*
TT (mm)	42.9 (40.0–45.9)	42.9 (39.3–46.0)	0.541
HMD (mm)	45.3 (42.4–48.5)	41.1 (38.5–44.9)	0.001*
DH (mm)	8.7 (6.6–10.9)	7.0 (5.3–9.1)	0.001*
WH (mm)	10.9 (9.8–12.7)	11.3 (10.2–13.1)	0.089
DSE (mm)	20.1 (16.6–22.5)	19.5 (16.5–21.6)	0.001*
DVC (mm)	7.1 (5.7–8.3)	6.8 (5.7–7.9)	0.033*

*DT* distance from skin to under surface of tongue, *TT* thickness of the thickest part of tongue body, *HMD* hyoid mental distance, *DH* depth of hyoid, *WH* width of hyoid, *DSE* distance from skin to epiglottis, *DVC* depth of the anterior combination of the vocal cords.

Data are shown as the median (*P*_25_*–P*_75_) deviation. All data were analyzed by Wilcoxon rank test, * *P* value < 0.05.

Seventy-four patients (71.15%) were classified as NDL group (CL < 2b), while 30 patients (28.85%) were classified as DL group (CL ≥ 2b) in direct laryngoscopy. The ROC curve analysis showed the AUC of each predictor. Sensitivity, specificity, PPV and NPV were shown in Table 4.

**Table 4:** Diagnostic validity profiles of effective predictors of DL.

Predictors	AUC (95% CI)	Sensitivity (95% CI)	Specificity (95% CI)	PPV	NPV
BMI (kg·m^−2^) > 22.9	0.645 (0.545–0.737)	0.533(0.346–0.712)	0.689(0.570–0.789)	0.410(0.260–0.578)	0.784(0.662–0.873)
Mouth opening(cm) < 4	0.562 (0.461–0.659)	0.300(0.154–0.496)	0.824(0.715–0.900)	0.409(0.215–0.633)	0.744(0.634–0.831)
Thyromental distance(cm) ≤ 6	0.642 (0.542–0.734)	0.300(0.154–0.496)	0.838(0.730–0.910)	0.429(0.226–0.656)	0.747(0.638–0.833)
Neck circumference(cm) > 35.75	0.723 (0.627–0.807)^a^	0.567(0.377–0.740)	0.811(0.700–0.889)	0.548(0.363–0.722)	0.822(0.711–0.898)
Modified Mallampati grade > 3	0.734 (0.638–0.807)^a^	0.500(0.317–0.683)	0.865(0.761–0.930)	0.600(0.389–0.782)	0.810(0.703–0.886)
DT (mm) < 14.7	0.508(0.408–0.607)	0.300(0.154–0.496)	0.581(0.461–0.693)	0.225(0.114–0.389)	0.672(0.542–0.781)
TT (mm) > 41.15	0.613(0.513–0.707)	0.833(0.645–0.937)	0.432(0.319–0.552)	0.373(0.261–0.500)	0.865(0.704–0.949)
HMD(mm) < 40.45	0.568(0.468–0.665)	0.067(0.012–0.235)	0.811(0.700–0.889)	0.125(0.022–0.396)	0.682(0.573–0.775)
DH(mm) > 5.2	0.506(0.406–0.605)	1.00(0.859–1.00)	0.125(0.070–0.239)	0.319(0.229–0.424)	1.00(0.655–1.00)
DSE(mm) > 21.25	0.868(0.788–0.926)^a^	0.800(0.607–0.916)	0.838(0.730–0.910)	0.667(0.489–0.809)	0.912(0.811–0.964)
DVC(mm) > 7.55	0.525(0.424–0.623)	0.433(0.260–0.623)	0.649(0.528–0.754)	0.333(0.196–0.503)	0.738(0.612–0.836)

*AUC* area under the curve, *PPV* positive predictive value, *NPV* negative predictive value, *BMI* body mass index, *DT* distance from skin to under surface of tongue, *TT* thickness of the thickest part of tongue body, *HMD* hyoid mental distance, *DH* depth of hyoid, *WH* width of hyoid, *DSE* distance from skin to epiglottis, *DVC* depth of the anterior combination of the vocal cords.

^a^AUC > 0.7 means that diagnostic value is high.

**Figure 3:**
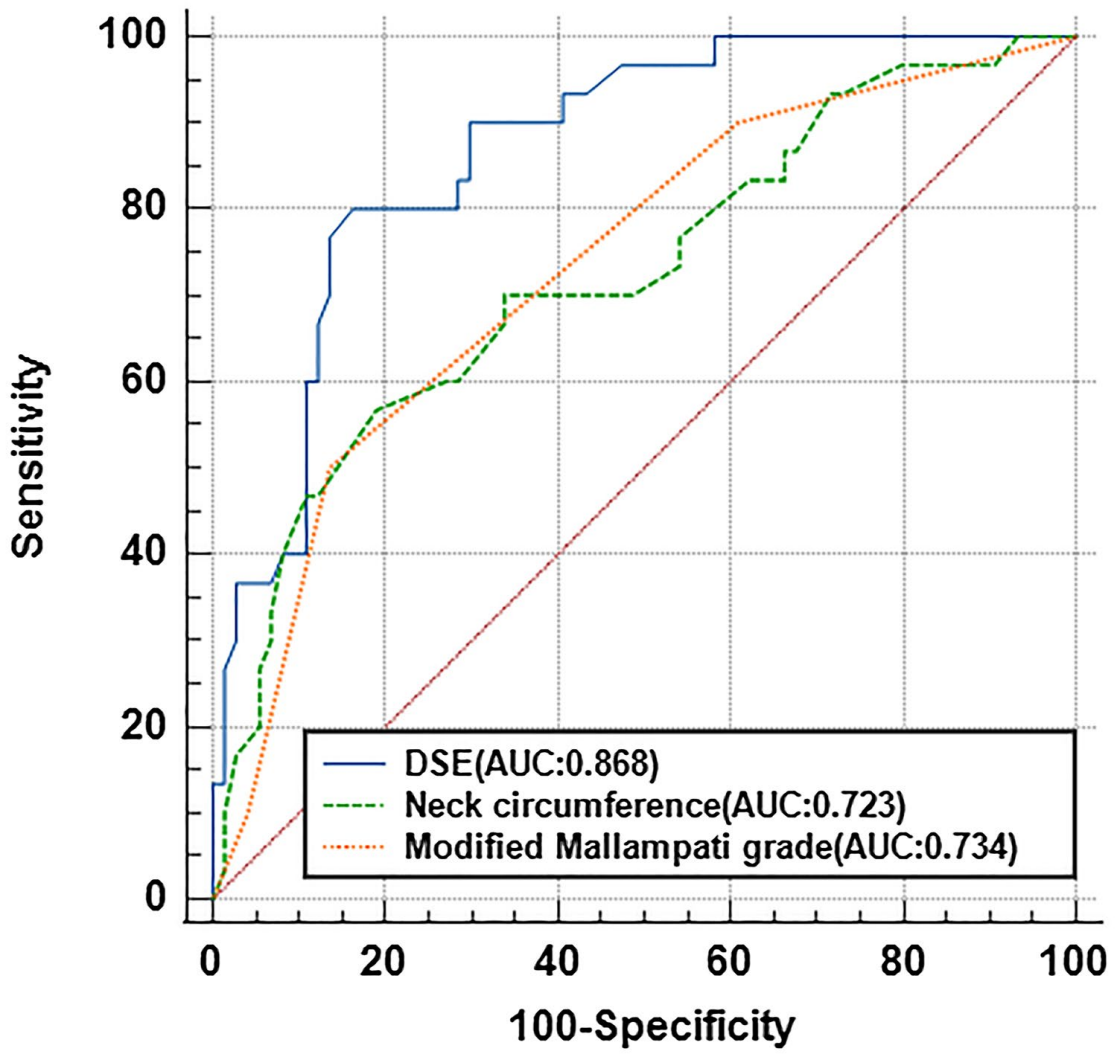
The ROC curves of DSE (blue line), neck circumference (green line) and modified Mallampati grade (red line), the AUC > 0.7 were showed. Asterisk: comparison with the AUC of the other 2 airway assessment tests, there were significant differences between DSE and other predictors (*P* < 0.05). Calculated by nonparametric test.

Table 2 showed there were statistical differences between NDL and DL groups in BMI, thyromental distance, neck circumference, modified Mallampati grade and DSE. However, Table 4 confirmed that only three variables of neck circumference, modified Mallampati grade and DSE might have diagnostic value in DL prediction (AUC was more than 0.7). The ROC curve of significant variables of neck circumference, modified Mallampati grade and DSE were demonstrated in Fig. 3.

The cut-off value of the DSE prediction criterion for DL determined by Youden index was 21.25 mm. The AUC of the DSE (AUC: 0.868) was bigger than other predictors. The sensitivity and specificity of DSE predicting DL were 80.0% (95% CI: 60.7–91.6%) and 83.8% (95% CI: 73.0–91.0%), respectively.

Hyoid bone is a bone structure of the upper airway and the width of it will not change after anaesthesia induction. In this study, the width of hyoid bone was 10.8 (9.8–12.3) mm and 11.2(10.1–12.5) mm before and after anaesthesia induction, respectively. There was no significant difference of hyoid bone between two states (*P* = 0.089 > 0.05), which showed good accuracy and repetitive stability of our results.

## Discussion

This study examined the changes of upper airway parameters before and after induction of anaesthesia by ultrasound device. The major findings of the study were (1) that the DT was deeper after induction of anaesthesia, while HMD, DH, DSE and the DVC were decreased, suggesting the propensity for the upper airway collapsibility was tongue drops close to hand palate and cartilaginous structures came-up with muscle relax, and (2) that data of the DSE measured by ultrasound device before anaesthesia may be a better predictor for DL.

The assessing devices for morphology changes of upper airway before and after induction of anaesthesia include rulers, radiography equipment, computerized tomography, magnetic resonance imaging and so on^20–22^. Compared with the aforementioned methods, ultrasound plays an important role in various diagnostic or therapeutic purposes in the operating theatre and critical care settings, especially the increasing use in airway management in the last decade^23^. In some studies^24,25^, ultrasound could be used for the care of pediatric and obstetric airway, confirmation of proper laryngeal mask airway position and evaluation of full stomach^26,27^. Emanuela Roldi et al^28^ found that ultrasound measurement of tracheal diameter could assist with the choice of the size of left double-lumen tube. To our knowledge, however, studies that involved ultrasound measurements for morphology changes of upper airway after anaesthesia induction were less reported. Our findings extend the application of this technique to managing airways undergoing general anaesthesia. This study indicated that it was relatively simple to identify the different airway structures with the ultrasound image. We also found that the propensity for the upper airway to collapse after muscular relaxation obtained by ultrasound was consistent with previous studies^4,29^, and similar to Simons JC^30^, this collapsibility of airway after induction of anaesthesia was stable. In all of these studies, the authors observed upper airway closing pressure (P_CLOSE_) and genioglossus muscle electromyography to endpoints^29–31^, while we visibly recorded changes of upper airway before and after anaesthesia induction by ultrasound device, it might show a high degree of accuracy by “your eyes”. Identification the changes in upper airway after anaesthesia induction is conducive to airway management, especially if upper airway collapsibility occurs in outdoor anaesthesia, such as ERCP and gastro-endoscope. Therefore, when it comes to this, the results of this study suggest that it is crucial to avoid tongue blocking laryngeal and restore muscle tension in the neck as soon as possible.

In this study, compared with that before anaesthesia, we found the DT was deeper, while HMD and DH were decreased, which could be partly explained by the decrease of related muscle activity and lung volume under anaesthesia induction^30^. On the one hand, the inhibitory effects on central drive to genioglossus activity in propofol anaesthesia will lead to loss of support on pharyngeal in humans^4^, the animal studies^32^ in cats also showed electromyographic activity of the genioglossus muscle and hypoglossal nerve activity decreased in during halothane anaesthesia. On the other hand, inspiratory and respiratory pump muscles inhibited after anaesthesia induction could also contribute to the observed changes in upper airway collapsibility. Lung volume decreased caused by such inhibitory effects on muscles after anaesthesia, could also result in upper airway collapsibility by virtue of longitudinal tension within the airway wall and a decreased caudal traction on the upper airway^33^.

In other recent studies, Weidong et al.^34^ performed sonographic measurements of mandibular condylar mobility to predict DL, and Yadav et al.^35^ showed ultrasound measurements of soft tissue thickness of the anterior neck and tongue thickness along with the clinical assessment of airway could be useful in predicting DL. Only one or two ultrasound parameters were recorded in these studies, while clinical screening tests and other seven ultrasonography parameters to compare the diagnosis value for DL were systematically collected in our study. We compared clinical screening tests including BMI, mouth opening, thyromental distance, neck circumference, modified Mallampati grade and six selected upper airway ultrasound parameters such as DT, TT, HMD, DH, DSE and the DVC. Our data demonstrated that the AUC of the DSE was 0.868 which was significantly bigger than the other(*P* < 0.05) predictors. Additionally, when the DSE exceeded 21.25 mm, the sensitivity and specificity of predicting DL were 80.0% (95% CI: 60.7–91.6%) and 83.8% (95% CI: 73.0–91.0%), respectively. Pinto^16^ and Martinez-Garcia^36^ also found that the DSE was associated to DL, but the cut-off values were not the same, which could be explained by small sample sizes and different areas where subjects came from. In our observational study, we expanded the sample size and compared the DSE measured by ultrasound device with other 11 indicators, which trying to get a more complete understanding of the DSE prediction DL. Increasing thickness at the level of the pre-epiglottic space could affect the ability to visualise the glottis with a Macintosh blade at direct laryngoscopy^19^. It could explain that a higher upwards concavity of the oropharyngeal curve would not overlap with pharyngo-glottic-tracheal curve, which lead to bad glottic visualization when both curves were not aligned with the visual axis^37^. The incidence of DL was 28.85% in our study, which was higher than others which defined CL grades ≥ 3 as DL^36^ (we defined DL as CL grades ≥ 2b), In our study, the incidence of DL was 28.85%, it was higher than other studies which defining CL grades ≥ 3 as DL^36^, (we defined CL grades ≥ 2b as DL), because some researchers believed that DL might have occurred on the condition of novice anaesthetists when the CL grade was 2b^17,19^.

Our study has several limitations. Potential selection bias might be existed since we excluded patients with obviously difficult intubation. On the one hand, it was a concern for the safety of the patients, on the other hand, we thought it might allow us to evaluate changes of upper airway in normal patients and use ultrasound measurements to predict unknown DL. The total time to measure all the sonographic variables was approximately 10 min in each patient before and after induction of anaesthesia, which was more time-consuming than other studies^34–36^. Our purposes were to identify the changes of upper airway via more variables, and screened out which variable might have the maximum correlation with the laryngoscopic view. Although seven distances from five ultrasound images were measured in our study, it was still difficult in fully displaying the upper airway changes during anaesthesia induction when compared with MRI and CT^20,21^, while they were more time-consuming and expensive. Furthermore, all measurements were performed within the images in store, not in real time, more data of upper airway structures should be collected during dynamic motion.

Finally, it has been suggested that the thickness of the base of the tongue (SBL) and hyoid mental distance with hyperextension of the head and subluxation of the mandible (DIMs) could effectively predict difficult mask ventilation, which may be caused by thick tongue could be displaced more posteriorly after anaesthesia, and reduced the air space also at the level of the oropharynx and hypopharynx limiting the airflow coming^11^. However, our study did not include difficult mask ventilation into the outcomes, because other studies suggested that mask ventilation could be facilitated by muscle relaxation after anaesthesia in adults^38,39^, it was quite controversial. Although our study found that parameters such as SBL and hyoid mental distance have no predictive value for difficult laryngoscopy, it was not discussed whether these ultrasound measurements could show values in difficult mask ventilation prediction, so we will further expand the sample size and include difficult mask ventilation as one of the outcomes in the follow-up research to obtain more complete results.

In summary, our study shown a new measurement process of upper airway after induction of anaesthesia. Our data measured by ultrasound device demonstrated that the propensity of upper airway collapsibility was tongue drops close to the hard palate and cartilaginous structures came-up after induction of anaesthesia. These results might provide a systematic investigation in upper airway, and we found the DSE assessed by ultrasound may be crucial to predict DL when compared with other indicators. Moreover, establishing a further prediction of difficult Laryngoscopy is required to reduce the incidence of the false negatives and the false positives, it can ensure patients’ safety and make the best use of resources.

## Supplementary Information

Supplementary Information.

## Data Availability

All data generated or analysed during this study are included in this published article [and its Supplementary Information files].
